# Hospitalizations for oral and oropharyngeal cancer in Brazil by the SUS: impacts of the covid-19 pandemic

**DOI:** 10.11606/s1518-8787.2023057004708

**Published:** 2023-05-11

**Authors:** Amanda Ramos da Cunha, Sofia Rafaela Maito Velasco, Fernando Neves Hugo, José Leopoldo Ferreira Antunes

**Affiliations:** I Universidade de São Paulo Faculdade de Saúde Pública Departamento de Epidemiologia São Paulo SP Brasil Universidade de São Paulo. Faculdade de Saúde Pública. Departamento de Epidemiologia. São Paulo, SP, Brasil; II Centro de Estudos, Pesquisa e Prática em APS e Redes Hospital Israelita Albert Einstein São Paulo SP Brasil Centro de Estudos, Pesquisa e Prática em APS e Redes. Hospital Israelita Albert Einstein. São Paulo, SP, Brasil; III Universidade Federal do Rio Grande do Sul Faculdade de Odontologia Departamento de Odontologia Preventiva e Social Porto Alegre RS Brasil Universidade Federal do Rio Grande do Sul. Faculdade de Odontologia. Departamento de Odontologia Preventiva e Social. Porto Alegre, RS, Brasil

**Keywords:** Mouth Neoplasms, Oropharyngeal Neoplasms, Hospital Care, Unified Health System, COVID-19, Pandemics

## Abstract

**OBJECTIVE:**

To analyze the impact of the different phases of the covid-19 pandemic on hospitalizations for oral (CaB) and oropharyngeal (CaOR) cancer in Brazil, carried out within the scope of the Brazilian Unified Health System (SUS).

**METHODS:**

We obtained data regarding hospital admissions due to CaB and CaOR between January 2018 and August 2021 from the SUS Hospital Information System, analyzing hospital admissions as rates per 100,000 inhabitants. We divided the pandemic (January 2020 to August 2021) and pre-pandemic (January 2018 to December 2019) periods into four-month periods, comparing the pandemic period rates with analogous rates for the pre-pandemic period – for Brazil, by macro-region and by a group of procedures performed during hospitalization. We also analyzed the impact of the pandemic on the average cost of hospitalizations, expressing the results in percentage change.

**RESULTS:**

Rates of hospitalization in the SUS due to CaB and CaOR decreased during the pandemic in Brazil. The most significant reduction occurred in the second four-month period of 2020 (18.42%), followed by decreases in the third four-month period of 2020 (17.76%) and the first and second four-month periods of 2021 (respectively, 14.64% and 17.07%), compared with 2019. The South and Southeast showed the most expressive and constant reductions between the different phases of the pandemic. Hospitalizations for clinical procedures suffered a more significant decrease than for surgical procedures. In Brazil, the average expenditure per hospitalization in the four-month pandemic periods was higher than in the reference periods.

**CONCLUSION:**

After more than a year of the pandemic’s beginning in Brazil, the SUS hospital care network for CaB and CaOR had yet to be re-established. The repressed demand for hospitalizations for these diseases, which have fast evolution, will possibly result in delays in treatment, negatively impacting the survival of these patients. Future studies are needed to monitor this situation.

## INTRODUCTION

Cancer currently occupies the second position among the diseases that cause the most deaths and years of life lost due to disability worldwide. According to the 2019 Global Burden of Disease Study, oral (CaB) and oropharyngeal (CaOR) cancers accounted for approximately 2.3% of new cases and 3.1% of deaths from all cancers in the world in 2019, which represents about 540,000 new cases and 313,000 deaths^1^. In Brazil, this subtype occupies the fifth position among the most frequent neoplasms in men^2^. Its incidence in Brazil, for both sexes, was estimated at 5.6 cases per 100,000 inhabitants by the Global Cancer Observatory 2020 (Globocan); it is the second highest rate in Latin America, lower only than the rate in Cuba^3^.

The treatment of CaB and CaOR involves surgery and/or radiotherapy associated, or not, with chemotherapy and depends on the hospital structure^4^. Surgeries, which tend to be highly complex, require hospitalization, and radio and chemotherapy treatments, as well as clinical treatments for complications or intercurrences, may also require hospital care. Invasive or advanced-stage lesions often involve mutilating surgical approaches, such as glossectomy and maxillectomy, of which possible consequences are orofacial deformities and functional deficits. Due to treatment sequelae, these patients may depend on hospital care, including recurrent and long-period hospitalizations. More than half of patients with head and neck cancer – a group that includes oral and oropharyngeal cancers – start treatment with lesions in advanced stages^5,6^ and, consequently, are more dependent on highly complex care and the hospital environment. Oral and oropharyngeal cancers are considered health problems with high economic and social impact, and, in general, the more complex the treatment required, the more expensive it becomes^7^. From 2008 to 2016, the Brazilian Unified Health System (SUS) spent around 500 million reais on hospitalizations to treat this subtype of cancer^8^.

The covid-19 pandemic has affected health systems worldwide, and care provision to cancer patients has also reflected this health crisis. Reduction in the routine activities of cancer care services and the number of surgeries, postponement of elective treatments and diagnostic procedures, and suspension of screening services were some of the impacts reported in the literature^9,10^. The fear of contamination by the new disease may also have kept symptomatic patients away from health services. Regarding CaB and CaOR, empirical evidence from the initial period of the pandemic indicated a reduction in the number of diagnostic procedures and hospitalizations in Brazil^11,12^, even in the face of the non-interruption of the operation of reference cancer services in the country. Timely diagnosis and treatment are critical factors for the survival of patients with CaB and CaOR^5,13,14^, and due to their potential to cause delays in the identification and treatment of these diseases, possible disruptions in the care network due to the pandemic need to be understood.

At the beginning of March 2022 – this study’s conduction period –, Brazil had more than 29 million confirmed cases and about 653,000 deaths by covid-19^15^. Since February 2020, when the first case of the disease was reported in the country, the pandemic has been characterized by different phases, with periods of resurgence and others of attenuation. Although national lockdown policies were not implemented, there was greater rigidity in local measures (municipal and state) of social distancing in periods of worsening pandemic^16^. During these periods, the Brazilian health system faced intense overload, which may have impacted the care for other diseases prevalent in the country. However, the impact of the breakdown of health services on care for other conditions, considering the different stages of the pandemic in Brazil, is not yet known. Surveillance of cancer care services is essential to maintain their effectiveness and to comprehend and mitigate the possible effects of the current health crisis in the care of this disease. Thus, this study aims to analyze the impact of covid-19 on hospitalizations for CaB and CaOR in the SUS, considering the different phases of the pandemic.

## METHODS

This study analyzed hospitalizations for CaB and CaOR (ICD-10 C00-C10), recorded in the SUS Hospital Information System (SIH-SUS) between January 2018 and August 2021. This information system consolidates and makes available – publicly and anonymously – data on all hospital admissions in Brazil within the scope of the SUS. To ensure that the most recent periods of the study did not reflect possible delays in the consolidation of data by the SIH – as hospitalizations can be processed in the SIH with some delay concerning the period in which they occurred –, information from the period of interest was retrieved from the databases from the months following that period (until November 2021). The SIH databases, made available by month and by Federation Unit (UF), were unified using the TabWin, a tabulation tool the Brazilian Ministry of Health offers.

We compared the months of the pandemic period (2020 and 2021) with their analogs in the reference period (2018 and 2019) to analyze the effect of the pandemic on hospital admissions for CaB and CaOR – the comparison of the pandemic years with the average of the years 2018 and 2019 was performed as a validation analysis of the comparison with the year 2019. We analyzed the study period by month and four-month period, assessing monthly and four-monthly hospitalizations using rates – dividing the number of hospitalizations in the month or the four months in each UF and macro-region by the number of inhabitants and multiplying by 100,000. We collected the number of hospitalizations by the state of residence and month of hospitalization and obtained the number of inhabitants in each UF/macro-region using population projections from the Brazilian Institute of Geography and Statistics (IBGE). We presented the comparison between the rates as a percentage of variation, obtained by the equation: [(rate of the pandemic period under analysis/rate of the comparable period in the reference year) -1] *100.

Each hospital admission recorded in the SIH is linked to the primary procedure performed in that hospitalization. We extracted the number of procedures per group from the SIH using the Procedure Group selection to understand the impact of the pandemic on the types of procedures performed on patients hospitalized for CaB and CaOR. The SUS classifies the procedures performed in their health services into eight groups. Groups 3 (Clinical Procedures) and 4 (Surgical Procedures) are the main ones for hospitalizations in which the primary diagnosis is CaB and CaOR. We analyzed the four-monthly number of procedures for these two groups using rates – dividing the number of clinical and surgical procedures (separately) in the four months in each macro-region by the number of inhabitants and multiplying by 100,000. We compared the 2021 and 2020 rates with 2019 as a percentage of change. Finally, we calculated the average expenditure per hospitalization to analyze the impact of the pandemic on the amounts spent on hospitalizations. We divided the total amount spent on hospitalizations due to CaB and CaOR (which included hospital and professional services) per four-month period by the number of hospitalizations in the same period, in each macro-region – both metrics obtained from the SIH. Then, we compared the mean value per hospitalization between the four months of the pandemic with its analogs in the reference period.

We presented this study’s results by UF and macro-region of Brazil: North, Northeast, Southeast, South, and Midwest. For the presentation by UF, we created maps with cartographic bases obtained through the IBGE website. All analyses employed the Stata 14.0 software.

## RESULTS

Hospitalization rates for CaB and CaOR in Brazil carried out by the SUS reduced in pandemic years compared with the reference years (2018 and 2019). For Brazil, the most significant reductions occurred in the second and third four-month periods of 2020 and the first and second ones of 2021, respectively, 18.42%, 17.76%, 14.64%, and 17.07% of reduction, considering the comparison with the rates of the analogous 2019 four-month periods. In these periods, a decrease occurred in the rates of the country’s five regions. The South presented the most expressive decreases (above 20%), while the Northeast region had the smallest reductions in the third four-month period of 2020 and the first and second four-month periods of 2021 – of less than 5%. Table 1 shows these results and the rates for each period.

**Table 1:** Four-month period rate of hospital admissions for oral and oropharyngeal cancer (per 100,000 inhabitants) by macro-region, and for Brazil, considering the pre-pandemic (2018 and 2019) and the pandemic (2020 and 2021) periods, and percentage difference between the periods.

Variable	2018–2019 Rate (average)	2019 Rate	2020 Rate	2021 Rate	2020/ 2018–2019 Variation (%)	2020/2019 Variation (%)	2021/ 2018–2019 Variation (%)	2021/2019 Variation (%)
1^st^ Four-month period								
North	1.18	1.13	1.06	0.96	-10.17	-5.9	-18.81	-14.95
Northeast	2.5	2.47	2.58	2.43	3.4	4.29	-2.51	-1.67
Southeast	3.92	3.92	3.68	3.05	-6.29	-6.18	-22.18	-22.09
South	5.4	5.64	5.41	4.42	0.09	-4.08	-18.15	-21.56
Midwest	3.15	3.03	3.32	2.95	5.13	9.59	-6.56	-2.6
**Brazil**	**3.23**	**3.24**	**3.21**	**2.76**	**-0.71**	**-0.88**	**-14.5**	**-14.64**
2^nd^ Four-month period								
North	1.12	1.03	0.95	0.93	-15.17	-7.4	-17.04	-9.44
Northeast	2.7	2.77	2.3	2.73	-14.87	-17.07	1.27	-1.35
Southeast	4.07	3.94	3.33	3.15	-18.25	-15.6	-22.69	-20.18
South	5.29	5.78	4.39	4.21	-17	-23.93	-20.53	-27.16
Midwest	3.34	3.47	2.89	3.07	-13.63	-16.79	-8.2	-11.56
**Brazil**	**3.3**	**3.4**	**2.77**	**2.82**	**-16.15**	**-18.42**	**-14.77**	**-17.07**
3^rd^ Four-month period								
North	0.99	1.02	0.92	-	-6.86	-9.59	-	-
Northeast	2.63	2.73	2.62	-	-0.33	-4.02	-	-
Southeast	4.01	4.08	3.34	-	-16.68	-18.01	-	-
South	5.37	6.03	4.6	-	-14.41	-23.68	-	-
Midwest	3.35	3.59	2.86	-	-14.57	-20.31	-	-
**Brazil**	**3.27**	**3.49**	**2.87**	-	**-12.28**	**-17.76**	-	-

**Figure 1:**
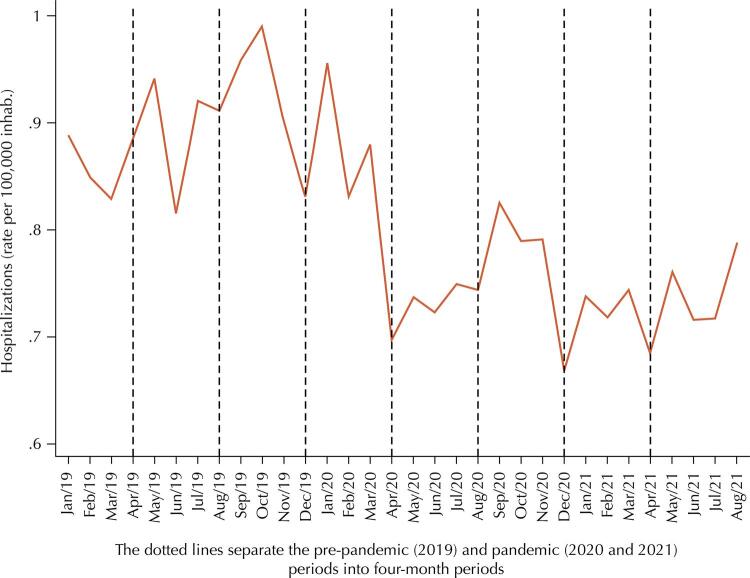
Monthly rate of hospital admissions (per 100,000 inhabitants) for oral and oropharyngeal cancer in Brazil, considering the pre-pandemic (2019) and pandemic (2020 and 2021) periods.

Figure 1 shows the monthly behavior of hospitalizations due to CaB and CaOR in Brazil, considering the period from January 2019 to August 2021. According to the Figure, the first significant drop in the rates of this indicator occurred in April 2020, i.e., at the end of the first four-month period of 2020. Since then, the indicators have remained below the level prior to that period. After this initial drop, September, October, and November 2020 showed slightly higher rates than the other months – however, still far from the pre-pandemic level. The hospitalization rates for the months of 2021 are lower than those for 2020. Despite the behavior suggestive of recovery in the last analyzed month of that year (August 2021), it is still far from the pre-pandemic level.

**Figure 2:**
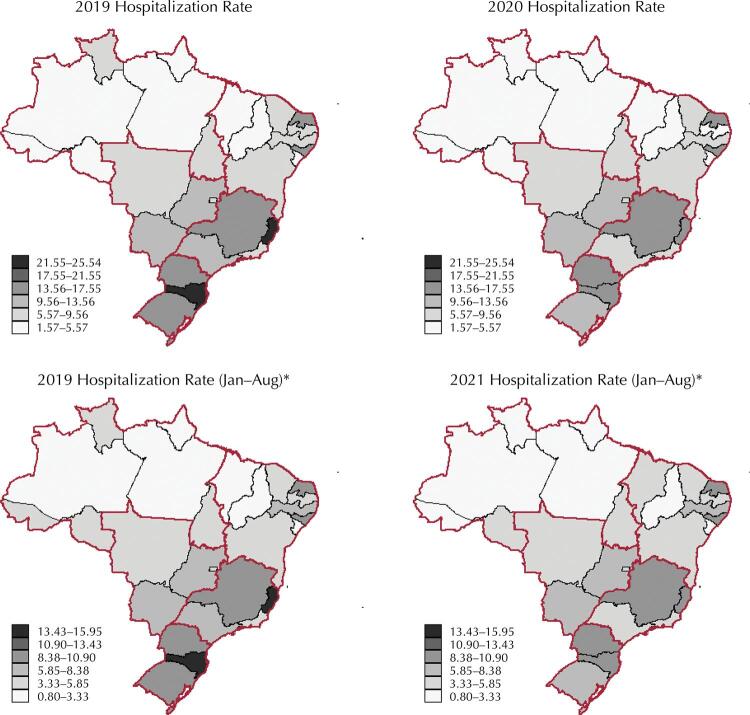
Annual rate of hospitalizations for oral and oropharyngeal cancer (per 100,000 inhabitants) by Federation Unit, considering the pre-pandemic (2019) and pandemic (2020 and 2021) periods.

Note: *The rate considers the period from January to August of the respective years for comparison purposes

Figure 2 shows the variation in hospitalization rates by CaB and CaOR by UF, comparing the years 2021 and 2020 with 2019. The results indicate the predominance of a pattern of rate reduction. In addition, they indicated that UFs with the highest hospitalization rates in the pre-pandemic period continued showing the highest rates in the pandemic years. In other words, the magnitude of the rates decreased in the pandemic years, but the pre-pandemic spatial pattern of this indicator remained: the lowest rates continued in the UFs in the North and Northeast regions; the highest in those of the South and Southeast regions.

The analysis of the groups of procedures performed on patients hospitalized for CaB and CaOR indicated that clinical procedures suffered a more significant reduction than surgical ones, comparing the 2021 and 2020 four-month periods with those of 2019. From the second four-month period of 2020 onward, the rates of both groups of procedures decreased in all periods. However, the decrease percentage was consistently more significant in rates for clinical procedures. Compared to the 2019 four-month periods, the most significant variation in the rates of clinical procedures occurred in the second four-month period of 2020 (-24.62%). In that same period, the variation in surgical procedures was -11.97% (Table 2). The clinical (Group 3) and surgical (Group 4) procedures together accounted for approximately 99.8% of all procedures performed on these patients in 2019, 2020, and 2021 (in 2019: Group 3 – 52.17% and Group 4 – 47.66%; in 2020: Group 3 – 49.81% and Group 4 – 49.97%; in 2021: Group 3 – 48.61% and Group 4 – 51.17%; data not shown).

**Table 2:** Four-month period rate of clinical and surgical procedures (per 100,000 inhabitants) in hospital admissions for oral and oropharyngeal cancer, by macro-region, and for Brazil, considering the pre-pandemic (2019) and pandemic (2020 and 2021) periods, and the percentage difference between periods.

Variable	Clinical Procedures (Group 3)					Surgical Procedures (Group 4)				
	
	Rate			Rate Variation (%)		Rate			Rate Variation (%)	
			
	2019	2020	2021	2020/2019	2021/2019	2019	2020	2021	2020/2019	2021/2019
1^st^ Four-month period										
North	0.5	0.48	0.44	-3.28	-11.76	0.63	0.57	0.52	-8.86	-17.5
Northeast	1.02	1.12	1.1	10.12	7.58	1.45	1.46	1.33	0.09	-8.5
Southeast	2.2	2.02	1.56	-8.29	-29.1	1.71	1.65	1.48	-3.37	-13.06
South	3.09	3.01	2.17	-2.54	-29.62	2.55	2.39	2.24	-6.33	-11.91
Midwest	1.43	1.75	1.52	22.09	5.98	1.57	1.57	1.43	-0.07	-8.88
**Brazil**	**1.65**	**1.68**	**1.36**	**1.72**	**-17.62**	**1.58**	**1.53**	**1.4**	**-3.47**	**-11.37**
2^nd^ Four-month period										
North	0.46	0.45	0.51	-2.29	9.31	0.57	0.5	0.42	-11.58	-25.71
Northeast	1.27	0.96	1.18	-24.73	-6.77	1.5	1.34	1.55	-10.59	3.13
Southeast	2.07	1.62	1.53	-22.01	-26.36	1.86	1.69	1.61	-9.44	-13.8
South	3.34	2.32	1.99	-30.64	-40.4	2.43	2.07	2.21	-14.89	-9.03
Midwest	1.64	1.29	1.45	-21.87	-11.87	1.82	1.6	1.61	-11.91	-11.3
**Brazil**	**1.76**	**1.32**	**1.33**	**-24.62**	**-24.27**	**1.64**	**1.44**	**1.48**	**-11.97**	**-9.55**
3^rd^ Four-month period										
North	0.49	0.44	-	-11.01	-	0.53	0.48	-	-8.26	-
Northeast	1.24	1.15	-	-7.41	-	1.49	1.47	-	-1.19	-
Southeast	2.18	1.71	-	-21.59	-	1.88	1.62	-	-13.69	-
South	3.61	2.3	-	-36.37	-	2.4	2.3	-	-4.2	-
Midwest	1.69	1.51	-	-10.57	-	1.9	1.35	-	-28.95	-
**Brazil**	**1.84**	**1.42**	-	**-22.89**	-	**1.64**	**1.45**	-	**-11.83**	-

**Figure 3:**
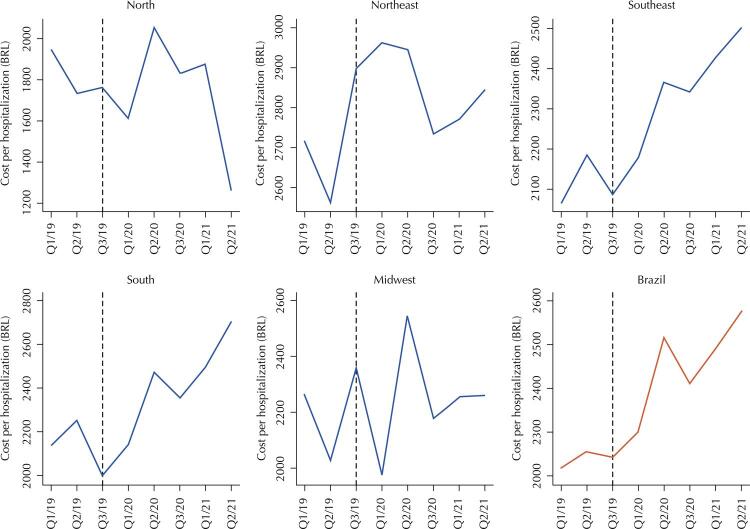
Average expenditure per hospitalization for oral and oropharyngeal cancer (in BRL), by four-month period, by macro-region, and for Brazil, considering the pre-pandemic (2019) and pandemic (2020 and 2021) periods.

Figure 3 presents the variation in average expenditure per hospitalization, considering the pre-pandemic and pandemic periods. In Brazil, the average spending per hospitalization in the four months of the pandemic was higher than in the comparable four-month period of the reference year (2019). This pattern also occurred in the South, Southeast, and Northeast regions – the latter had the third four-month period of 2020 as an exception, whose average expenditure per hospitalization was lower than in the same period in 2019. The North and Midwest showed a different behavior, with some four-month periods of the pandemic period having an average cost per hospitalization higher than in the pre-pandemic period, but others with the opposite situation (Figure 3).

In general, the analyses considering 2019 as a reference showed similar results to the validation analysis, which considered 2018 and 2019 average values as a reference.

## DISCUSSION

This study showed that hospitalizations for CaB and CaOR carried out by the SUS decreased in all phases of the pandemic in Brazil. The first and most crucial reduction occurred in the second four-month period of 2020 when the pandemic began to intensify in the country, prompting local governments to institute social distancing measures to contain the so-called first wave. From then on – until the end of the study period – even though there were periods with a less expressive reduction than that experienced in the second four-month period of 2020, hospital admissions for CaB and CaOR carried out by the SUS no longer returned to their pre-pandemic level. Identifying a sustained reduction in these rates is one of the main results of this analysis. By investigating the impact of the pandemic on hospital admissions for CaB and CaOR within the public health system in Brazil, this investigation provided evidence of the magnitude and distribution of the problem and can contribute to mitigating it.

Since they are considered essential, the SUS’s hospital and cancer care services remained in operation during the pandemic in Brazil. However, maintaining these services did not prevent the health crisis triggered by covid-19 from affecting hospital capacity for this disease, as indicated by the results of this article and research carried out in the initial period of the pandemic^11,17^. Possible reasons for this effect are diverse and may be related to the overload of the hospital structure due to the influx of cases of covid-19 requiring hospitalization, the concern to guarantee hospital beds for these cases, the lack of professionals, supplies, and/or equipment – depleted or reallocated for covid-19 assistance –, to the social consequences of the social distancing measures, and the economic crisis arising from the health crisis and the fear of the patients themselves of being infected by the new virus, thus choosing not to seek the healthcare services^18,19^, among others. Other countries, such as India, Spain, and the United Kingdom, also reported reductions in the capacity of the hospital system to treat cases of head and neck cancer due to the pandemic scenario^20^. In Spain, a study carried out in 44 hospital services indicated that 45.5% of them had to suspend oncological surgeries considered high priority in patients with head and neck cancer at some point during the pandemic^21^. However, these results are not directly comparable to those of this study – despite the similarity of the theme, the outcomes under analysis are different.

The reduction in cancer hospitalizations may still indicate, in part, postponement of treatment due to medical decisions to balance risks, as cancer patients have worse outcomes when affected by covid-19 than the general population^23,24^. However, specifically for CaB and CaOR, this is not the recommended situation. A statistical modeling study estimated that, in the pandemic context, cases of CaB and CaOR would be among the types most favored by immediate treatment, compared with postponement^25^. In updated recommendations for the period of the covid-19 pandemic, the European Society for Medical Oncology indicates that mouth and oropharynx tumors at the T1 stage should already be a high/medium priority for primary surgery, and all other ones should be a high priority^26^. Therefore, we believe that the reduction in hospitalizations found in this analysis is not essentially reflecting programmed delays in the treatment of neoplasms. The results may represent more serious situations, such as (1) barriers to accessing hospital treatment during the pandemic; and (2) impacts of the pandemic on the diagnosis of these diseases, with under-identifying cases that should be admitted to the hospital network for treatment. Decreased numbers of diagnostic procedures for oral cancer occurred in Brazil^12,27,28^. The reduction in hospitalizations due to underdiagnosis is a likely and worrying scenario, as cases not diagnosed during this period may arrive at hospital services with tumors at a more advanced stage, with a lower survival chance^5,13,14,29^.

This study analyzed the phases of the pandemic by four months and, considering the variation in the weekly number of deaths in the country since the notification of the first case (in February 2020)^15^, assumed that the first four-month period of 2020 represented the initial period of the pandemic in Brazil; the second represented the first wave; the third, the first period of attenuation of social distancing measures; and finally, the two four-month periods of 2021 represented the second wave of the pandemic. The first and most crucial drop in hospital admissions for CaB and CaOR within the scope of the SUS occurred in the first wave phase, when the health system faced its first collapse, with the depletion of material and human resources and structural overload due to the intense spread of covid-19 across the country. This was also the phase in which local governments implemented the most restrictive social distancing measures^16^. The reductions identified in the first wave phase are, therefore, compatible with the disorderly character of that period. However, although there were slight recoveries compared with the first wave phase, none of the subsequent ones showed a return of rates to pre-pandemic levels. The maintenance of these reductions more than a year after the start of the pandemic suggests that the impact of this health crisis on hospital care for CaB and CaOR was not a momentary situation, resulting only from an initial adaptation of care to a new and atypical context. The impact of this sustained reduction in mortality/survival from these neoplasms in the short and medium term is still uncertain and needs to be monitored, mainly because these are subtypes of cancer that, in regular times, are already too lethal – the average five-year survival is 50%^5^.

This study also identified that the reduction in hospital admissions carried out by the SUS varied in magnitude in the different macro-regions of the country. The North and, above all, the Northeast were the regions that showed the smallest reductions in hospitalization rates. On the other hand, the South and Southeast regions showed more significant and constant reductions during the different phases of the pandemic. Brazil’s South and Southeast regions have the best indicators of access to health services^30^, including higher rates of procedures for diagnosing oral cancer^12^. Due to the more significant provision of health services in these regions, more malignant lesions are likely diagnosed and, proportionally, more in the initial stages. As cancer diagnosis was an area greatly affected by the pandemic in the country, regions that performed more of these procedures possibly showed more significant reductions in hospitalizations. Furthermore, cases in initial staging are less likely to have been prioritized for treatment by the health system in the context of a health crisis since, given the limited supply of hospital structure for treatment, more advanced and urgent cases are expectedly prioritized.

The South and Southeast regions showed a notable increase in the mean value per hospitalization for CaB and CaOR, starting from the first wave phase – for the other regions, this trend was not so clear. We hypothesize that the South and Southeast have a higher possibility of organizing the flow of care in periods of limited resources through selective screening, including telemedicine, monitoring cases, and prioritizing more serious situations – which require more costly interventions. However, the pattern of expenditures in these regions may also reflect a decrease in the diagnosis of lesions at an earlier stage, which requires less invasive and costly treatments. Finally, clinical procedures linked to hospitalizations decreased more than surgical procedures in all pandemic phases, starting from the first wave phase. Guidelines for managing CaB and CaOR during the pandemic did not advocate replacing surgery with other treatment modalities. We believe the present results indicate that clinical demands are more likely to correspond to an elective and postponable or manageable need in an outpatient setting than surgical demands.

This study has several limitations that must be considered when interpreting its results. The SIH covers hospitalizations carried out by the SUS and does not consider those occurring within the scope of the supplementary network (private hospitals and those with health insurance agreements). Therefore, this study does not present an overview of all hospitalizations due to CaB and CaOR that occurred in the country. Data from the 2019 National Health Survey indicate that approximately 71.5% of the Brazilian population does not have a health insurance plan^31^, thus depending on the SUS for care/hospitalization needs. In addition, oral cancer is an outcome associated with socioeconomic conditions: individuals in more vulnerable situations – and who, consequently, depend more on public health services – are at increased risk of developing this disease^32^. Therefore, despite analyzing only data linked to the SUS, this article includes a significant portion of the population affected by this disease. Considering the universal nature of the SUS and the impossibility of accurately equating the proportion of patients with CaB and CaOR using health insurance plans in the hospital environment, it is noteworthy that the rates presented were calculated using the total population as a denominator.

The hospitalization rates presented here may account for more than one hospital admission of the same patient for the same cause, as the SIH provides consolidated data; therefore, these duplications cannot be identified and excluded. However, this is not a critical limitation for this analysis, which aimed to measure reductions in the provision of hospital services possibly associated with the pandemic without considering aspects related to individual risks. Furthermore, we highlight that analyzing the data by macro-region and using the four months to represent the pandemic phases – with standardization of this division for all regions – disregards the heterogeneity in the space-time patterns of dissemination present in the course of the pandemic in Brazil. Future studies that consider local characteristics of the dynamics of the pandemic in the country may be necessary.

This study revealed that, after more than a year of the beginning of the pandemic in Brazil, the hospital care network for CaB and CaOR of the SUS had not yet been re-established. It also showed evidence that, during the pandemic, the hospital network prioritized more serious cases of CaB and CaOR. The hospitalizations reductions may reflect barriers to access to hospital treatment due to the pandemic’s overload and disruption of this sector. The repressed demand for hospitalizations for cases of CaB and CaOR, which are rapidly evolving diseases, will be (and already is) related to delays in starting treatment, with a negative impact on the survival of these patients – a situation that will worsen proportionally to the time this breakdown lasts. This study highlights the need for health management attention to the CaB and CaOR care network, as this, in addition to the immediate need to resume its care capacity, will probably need to expand it in the short term to mitigate the probable damage caused by the pandemic in the care of these diseases and prevent this disruption from increasing the mortality.
