# Supplementary material for: Hospitalizations for oral and oropharyngeal cancer in Brazil by the SUS: impacts of the covid-19 pandemic
Source: Rev Saude Publica. 2023 May 11;57(Suppl 1):3s. doi: 10.11606/s1518-8787.2023057004708 (PMC10185317; doi:10.11606/s1518-8787.2023057004708)
Supplement: Translation

# Translation: Hospitalizações por câncer bucal e orofaríngeo no Brasil pelo SUS: impactos da pandemia de covid-19

**Authors:** Amanda Ramos da Cunha, Sofia Rafaela Maito Velasco, Fernando Neves Hugo, José Leopoldo Ferreira Antunes

**Affiliations:** I Brasil Universidade de São Paulo. Faculdade de Saúde Pública. Departamento de Epidemiologia. São Paulo, SP, Brasil; II Brasil Centro de Estudos, Pesquisa e Prática em APS e Redes. Hospital Israelita Albert Einstein. São Paulo, SP, Brasil; III Brasil Universidade Federal do Rio Grande do Sul. Faculdade de Odontologia. Departamento de Odontologia Preventiva e Social. Porto Alegre, RS, Brasil

**Keywords:** Neoplasias Bucais, Neoplasias Orofaríngeas, Hospitalização, Sistema Único de Saúde, COVID-19, Pandemias

## Abstract

**OBJETIVO:**

Analisar o impacto das diferentes fases da pandemia de covid-19 sobre as hospitalizações por câncer bucal (CaB) e de orofaringe (CaOR) no Brasil, realizadas no âmbito do Sistema Único de Saúde (SUS).

**MÉTODOS:**

Os dados quanto às internações hospitalares por CaB e CaOR, entre janeiro de 2018 e agosto de 2021, foram obtidos no Sistema de Informações Hospitalares do SUS. As internações foram analisadas sob a forma de taxas por 100 mil habitantes. Os períodos de pandemia (janeiro de 2020 a agosto de 2021) e pré-pandemia (janeiro de 2018 a dezembro de 2019) foram divididos em quadrimestres; as taxas quadrimestrais do período pandêmico foram comparadas às taxas análogas do período pré-pandemia – para o Brasil, por macrorregião e por grupo de procedimentos realizados na internação. O impacto da pandemia sobre o valor médio das internações também foi analisado. Os resultados foram expressos em variação percentual.

**RESULTADOS:**

As taxas de internação hospitalar no SUS por CaB e CaOR reduziram durante a pandemia no Brasil. Em comparação com os quadrimestres de 2019, a maior redução foi identificada no segundo quadrimestre de 2020 (18,42%), seguida das reduções do terceiro quadrimestre de 2020 (17,76%) e do primeiro e segundo quadrimestres de 2021 (respectivamente, 14,64% e 17,07%). Sul e Sudeste apresentaram as reduções mais expressivas e constantes entre as diferentes fases da pandemia. As internações para procedimentos clínicos sofreram maior redução do que para procedimentos cirúrgicos. No Brasil, o gasto médio por internação nos quadrimestres da pandemia foi maior do que nos quadrimestres de referência.

**CONCLUSÃO:**

Após mais de um ano do início da pandemia no Brasil, a rede hospitalar de cuidado ao CaB e CaOR do SUS ainda não tinha se restabelecido. A demanda reprimida de hospitalizações por essas doenças, que são de rápida evolução, possivelmente resultará em atrasos para tratamento, com impacto negativo para a sobrevida desses pacientes; futuros estudos são necessários para monitorar essa situação.

## INTRODUÇÃO

O câncer ocupa, atualmente, a segunda posição entre as doenças que mais causam mortes e anos de vida perdidos por incapacidade no mundo. De acordo com Global Burden of Disease Study de 2019, os cânceres bucal (CaB) e de orofaringe (CaOR) foram responsáveis por cerca de 2,3% dos casos novos e 3,1% dos óbitos ocorridos por todos os cânceres no mundo no ano em questão, o que representa cerca 540 mil novos casos e 313 mil óbitos^1^. No Brasil, esse subtipo ocupa a quinta posição entre as neoplasias mais incidentes nos homens^2^. Sua incidência no Brasil, para ambos os sexos, foi estimada em 5,6 casos a cada 100 mil pessoas pelo Global Cancer Observatory 2020 (Globocan) e é a segunda taxa mais alta da América Latina, menor apenas do que a taxa de Cuba^3^.

O tratamento dos CaB e CaOR envolve cirurgia e/ou radioterapia associadas, ou não, à quimioterapia e depende de estrutura hospitalar^4^. As cirurgias, que costumam ser de alta complexidade, requerem internações hospitalares e os tratamentos rádio e quimioterápico, bem como tratamentos clínicos de complicações ou intercorrências também podem exigir hospitalizações. Lesões invasivas ou com estadiamento avançado muitas vezes envolvem abordagens cirúrgicas mutiladoras, por exemplo, glossectomia e maxilectomia, das quais deformidades orofaciais e déficits funcionais são possíveis consequências; esses pacientes podem depender da assistência hospitalar, incluindo a internação nesses ambientes de maneira recorrente e por longos períodos, devido às sequelas do tratamento. Mais da metade dos pacientes com câncer de cabeça e pescoço – grupo no qual estão incluídos os cânceres bucal e de orofaringe – iniciam o tratamento com lesões em estágios avançados^5,6^ e, consequentemente, são mais dependentes de cuidados de alta complexidade e do ambiente hospitalar. Os cânceres bucal e de orofaringe são considerados agravos à saúde de alto impacto econômico e social e, de modo geral, quanto mais complexo o tratamento exigido, mais caro ele se torna^7^. De 2008 a 2016, o Sistema Único de Saúde do Brasil (SUS) gastou cerca de 500 milhões de reais em internações hospitalares para tratamento desse subtipo de câncer^8^.

A pandemia por covid-19 afetou os sistemas de saúde no mundo todo e a prestação de cuidados em pacientes com câncer também vem refletindo essa crise sanitária. Redução nas atividades de rotina dos serviços de atenção ao câncer e no número de cirurgias, adiamento de tratamentos eletivos e de procedimentos diagnósticos e suspensão de serviços de rastreamento foram alguns dos impactos reportados na literatura^9,10^. O receio de contaminação pela nova doença também pode ter afastado pacientes sintomáticos dos serviços de saúde. Em relação ao CaB e CaOR, evidências empíricas do período inicial da pandemia indicaram redução no número de procedimentos de diagnóstico e nas hospitalizações no Brasil^11,12^, mesmo diante da não interrupção do funcionamento dos serviços de referência em câncer no país. Diagnóstico e tratamento em tempo oportuno são fatores-chave para a sobrevida dos pacientes com CaB e CaOR^5,13,14^ e, por seu potencial de causar atrasos na identificação e no tratamento dessas doenças, possíveis desestruturações na rede de cuidados devido à pandemia precisam ser compreendidas.

No início de março 2022 – período de realização deste estudo – o Brasil contabilizava mais de 29 milhões de casos confirmados e cerca de 653 mil óbitos por covid-19^15^. Desde fevereiro de 2020, quando foi notificado o primeiro caso da doença no país, a pandemia foi caracterizada por diferentes fases, com períodos de recrudescimento e outros de atenuação. Apesar de não terem sido implementadas políticas nacionais de *lockdown*, houve maior rigidez nas medidas locais (municipais e estaduais) de distanciamento social nos períodos de agravamento da pandemia^16^. Nesses períodos, o sistema de saúde brasileiro enfrentou intensa sobrecarga, que pode ter refletido no cuidado às outras doenças prevalentes no país. Contudo, o impacto da desestruturação dos serviços de saúde na atenção a outros agravos, considerando as diferentes fases da pandemia no Brasil, ainda não é conhecido. A vigilância dos serviços de atenção ao câncer é fundamental para a manutenção da sua efetividade e para equacionar e mitigar os possíveis efeitos da atual crise sanitária no cuidado a essa doença. Desse modo, o objetivo deste trabalho é analisar o impacto da covid-19 nas hospitalizações por CaB e CaOR no SUS, considerando as diferentes fases da pandemia.

## MÉTODOS

Este estudo analisou as hospitalizações por CaB e CaOR (CID-10 C00-C10), registradas no Sistema de Informações Hospitalares do SUS (SIH-SUS), que ocorreram entre janeiro de 2018 a agosto de 2021. Esse sistema de informação consolida e disponibiliza, de maneira pública e anônima, dados de todas as internações hospitalares realizadas no Brasil no âmbito do SUS. Para garantir que os períodos mais recentes do estudo não refletissem possíveis atrasos na consolidação dos dados pelo SIH – pois as internações podem ser processadas no SIH com algum atraso em relação ao período em que ocorreram –, foram resgatadas informações do período de interesse nos bancos de dados dos meses subsequentes a esse período (até novembro de 2021). Os bancos de dados do SIH, que são disponibilizados por mês e por Unidade da Federação (UF), foram unificados por meio da ferramenta de tabulação TabWin, disponibilizada pelo Ministério da Saúde do Brasil.

Para analisar o efeito da pandemia sobre as internações hospitalares por CaB e CaOR, os meses do período pandêmico (2020 e 2021) foram comparados com seus análogos no período de referência (2018 e 2019) – a comparação dos anos pandêmicos com a média dos anos 2018 e 2019 foi feita como análise de validação da comparação com o ano de 2019. O período do estudo foi analisado por mês e por quadrimestre. As internações mensais e quadrimestrais foram analisadas sob a forma de taxas – o número de internações no mês ou no quadrimestre em cada UF e macrorregião foi dividido pelo número de habitantes e multiplicado por 100 mil. As internações foram coletadas por UF de residência e pelo mês de internação. O número de habitantes em cada UF/macrorregião foi obtido por meio das projeções populacionais do Instituto Brasileiro de Geografia e Estatística (IBGE). A comparação entre as taxas foi apresentada como percentual de variação, obtido por meio da equação: [(taxa do período pandêmico sob análise/taxa do período análogo em ano de referência) -1] *100.

Cada admissão hospitalar registrada no SIH está vinculada ao procedimento principal realizado naquela hospitalização. Para compreensão do impacto da pandemia nos tipos de procedimentos realizados nos pacientes internados por CaB e CaOR, a quantidade de procedimentos, por grupo, foi extraída do SIH, por meio da seleção Grupo de Procedimento. O SUS classifica os procedimentos realizados em seus serviços de saúde em oito grupos. Os grupos 3 (Procedimentos Clínicos) e 4 (Procedimentos Cirúrgicos) são os principais em internações nas quais o diagnóstico principal é CaB e CaOR. A quantidade quadrimestral dos procedimentos desses dois grupos foi analisada sob a forma de taxas: o número de procedimentos clínicos e cirúrgicos (separadamente) no quadrimestre, em cada macrorregião, foi dividido pelo número de habitantes e multiplicado por 100 mil. A comparação entre as taxas de 2021 e 2020 com 2019 foi apresentada como percentual de variação. Por fim, para análise do impacto da pandemia sobre os valores gastos nas hospitalizações, foi calculado o gasto médio por hospitalização: o valor total das internações por CaB e CaOR (que incluiu serviços hospitalares e profissionais), por quadrimestre, foi dividido pelo número de internações no mesmo período – as duas métricas foram obtidas no SIH. O valor médio por internação foi então comparado entre os quadrimestres da pandemia e os seus análogos do período de referência.

Os resultados deste estudo foram apresentados por UF e por macrorregião do Brasil – Norte, Nordeste, Sudeste, Sul e Centro-Oeste. Para apresentação por UF, foram criados mapas cujas bases cartográficas foram obtidas por meio do website do IBGE. Todas as análises foram realizadas no software Stata 14.0.

## RESULTADOS

As taxas de internação hospitalar por CaB e CaOR no Brasil, realizadas no âmbito do SUS, reduziram nos anos pandêmicos em comparação com os anos de referência (2018 e 2019). Para o Brasil, as maiores reduções foram identificadas no segundo e terceiro quadrimestres de 2020 e no primeiro e segundo quadrimestres de 2021: respectivamente, 18,42%, 17,76%, 14,64% e 17,07% de redução, considerando a comparação com as taxas dos quadrimestres análogos do ano de 2019. Nesses períodos, houve redução nas taxas das cinco regiões do país e o Sul foi a que apresentou as diminuições mais expressivas (acima de 20%). Já a região Nordeste foi a que apresentou as menores diminuições no terceiro quadrimestre de 2020 e no primeiro e segundo quadrimestres de 2021 – de menos de 5%. Esses resultados e as taxas de cada período estão expostos na Tabela 1.

**Tabela 1:** Taxa quadrimestral de internações hospitalares por câncer bucal e de orofaringe (a cada 100 mil habitantes), por macrorregião e para o Brasil, considerando período pré-pandemia (2018 e 2019) e período pandêmico (2020 e 2021), e porcentagem de diferença entre os períodos.

Variável	Taxa 2018–2019 (média)	Taxa 2019	Taxa 2020	Taxa 2021	Variação (%) 2020/ 2018–2019	Variação (%) 2020/2019	Variação (%) 2021/ 2018–2019	Variação (%) 2021/2019
1º Quadrimestre								
Norte	1,18	1,13	1,06	0,96	-10,17	-5,9	-18,81	-14,95
Nordeste	2,5	2,47	2,58	2,43	3,4	4,29	-2,51	-1,67
Sudeste	3,92	3,92	3,68	3,05	-6,29	-6,18	-22,18	-22,09
Sul	5,4	5,64	5,41	4,42	0,09	-4,08	-18,15	-21,56
C.Oeste	3,15	3,03	3,32	2,95	5,13	9,59	-6,56	-2,6
**Brasil**	**3,23**	**3,24**	**3,21**	**2,76**	**-0,71**	**-0,88**	**-14,5**	**-14,64**
2º Quadrimestre								
Norte	1,12	1,03	0,95	0,93	-15,17	-7,4	-17,04	-9,44
Nordeste	2,7	2,77	2,3	2,73	-14,87	-17,07	1,27	-1,35
Sudeste	4,07	3,94	3,33	3,15	-18,25	-15,6	-22,69	-20,18
Sul	5,29	5,78	4,39	4,21	-17	-23,93	-20,53	-27,16
C.Oeste	3,34	3,47	2,89	3,07	-13,63	-16,79	-8,2	-11,56
**Brasil**	**3,3**	**3,4**	**2,77**	**2,82**	**-16,15**	**-18,42**	**-14,77**	**-17,07**
3º Quadrimestre								
Norte	0,99	1,02	0,92	-	-6,86	-9,59	-	-
Nordeste	2,63	2,73	2,62	-	-0,33	-4,02	-	-
Sudeste	4,01	4,08	3,34	-	-16,68	-18,01	-	-
Sul	5,37	6,03	4,6	-	-14,41	-23,68	-	-
C.Oeste	3,35	3,59	2,86	-	-14,57	-20,31	-	-
**Brasil**	**3,27**	**3,49**	**2,87**	-	**-12,28**	**-17,76**	-	-

A Figura 1 exibe o comportamento mensal das internações por CaB e CaOR no Brasil, considerando o período de janeiro de 2019 a agosto de 2021. Identifica-se que a primeira e principal queda nas taxas desse indicador ocorreu em abril de 2020, ou seja, no final do primeiro quadrimestre de 2020. Desde então, os indicadores mantiveram-se abaixo do patamar anterior a esse período. No período posterior a essa queda inicial, os meses setembro, outubro e novembro de 2020 exibiram taxas um pouco maiores do que os demais meses – contudo, ainda distantes do nível pré-pandemia. As taxas de internação dos meses de 2021 são menores do que as taxas do ano de 2020; há um comportamento sugestivo de recuperação no último mês analisado desse ano (agosto de 2021), mas que, novamente, ainda é distante do patamar pré-pandêmico.

**Figura 1:** Taxa mensal de internações hospitalares (a cada 100 mil habitantes) por câncer bucal e de orofaringe no Brasil, considerando período pré-pandemia (2019) e período pandêmico (2020 e 2021).

A Figura 2 apresenta a variação das taxas de internação por CaB e CaOR por UF, considerando a comparação dos anos de 2021 e 2020 com 2019. Os resultados indicaram a predominância de um padrão de redução das taxas. Além disso, indicaram que as UF que apresentavam as maiores taxas de hospitalização no período pré-pandêmico continuaram apresentando as maiores taxas nos anos pandêmicos. Ou seja, a magnitude das taxas diminuiu nos anos de pandemia, mas o padrão espacial pré-pandemia desse indicador se manteve: as menores taxas continuaram nas UF das regiões Norte e Nordeste; as maiores, nas UF das regiões Sul e Sudeste.

**Figura 2:** Taxa anual de internações hospitalares por câncer bucal e de orofaringe (a cada 100 mil habitantes), por Unidade da Federação, considerando período pré-pandemia (2019) e período pandêmico (2020 e 2021).

Nota: *A taxa considera o período de janeiro a agosto dos respectivos anos, para fins de comparação

As análises dos grupos de procedimentos realizados nos pacientes internados por CaB e CaOR indicaram que os procedimentos clínicos sofreram maior redução do que os procedimentos cirúrgicos, considerando a comparação dos quadrimestres de 2021 e 2020 com os de 2019. A partir do segundo quadrimestre de 2020, houve queda nas taxas de ambos os grupos de procedimentos, em todos os quadrimestres; contudo, a porcentagem de diminuição foi sempre maior nas taxas dos procedimentos clínicos. Em comparação com os quadrimestres de 2019, a maior variação nas taxas dos procedimentos clínicos ocorreu no segundo quadrimestre de 2020 (-24,62%). Nesse mesmo período, a variação nos procedimentos cirúrgicos foi de -11,97% (Tabela 2). Os procedimentos clínicos (Grupo 3) e cirúrgicos (Grupo 4) somados representaram cerca de 99,8% de todos os procedimentos realizados nesses pacientes em 2019, 2020 e 2021 (em 2019: Grupo 3 – 52,17% e Grupo 4 – 47,66%; em 2020: Grupo 3 – 49,81% e Grupo 4 – 49,97%; em 2021: Grupo 3 – 48,61% e Grupo 4 – 51,17%; dados não apresentados).

**Tabela 2:** Taxa quadrimestral de realização de procedimentos clínicos e cirúrgicos (a cada 100 mil habitantes) em internações hospitalares por câncer bucal e de orofaringe, por macrorregião e para o Brasil, considerando período pré-pandemia (2019) e período pandêmico (2020 e 2021), e porcentagem de diferença entre os períodos.

Variável	Procedimentos Clínicos (Grupo 3)					Procedimentos Cirúrgicos (Grupo 4)				
	
	Taxa			Variação das Taxas (%)		Taxa			Variação das Taxas (%)	
			
	2019	2020	2021	2020/2019	2021/2019	2019	2020	2021	2020/2019	2021/2019
1º Quadrimestre										
Norte	0,5	0,48	0,44	-3,28	-11,76	0,63	0,57	0,52	-8,86	-17,5
Nordeste	1,02	1,12	1,1	10,12	7,58	1,45	1,46	1,33	0,09	-8,5
Sudeste	2,2	2,02	1,56	-8,29	-29,1	1,71	1,65	1,48	-3,37	-13,06
Sul	3,09	3,01	2,17	-2,54	-29,62	2,55	2,39	2,24	-6,33	-11,91
C.Oeste	1,43	1,75	1,52	22,09	5,98	1,57	1,57	1,43	-0,07	-8,88
Brasil	1,65	1,68	1,36	1,72	-17,62	1,58	1,53	1,4	-3,47	-11,37
2º Quadrimestre										
Norte	0,46	0,45	0,51	-2,29	9,31	0,57	0,5	0,42	-11,58	-25,71
Nordeste	1,27	0,96	1,18	-24,73	-6,77	1,5	1,34	1,55	-10,59	3,13
Sudeste	2,07	1,62	1,53	-22,01	-26,36	1,86	1,69	1,61	-9,44	-13,8
Sul	3,34	2,32	1,99	-30,64	-40,4	2,43	2,07	2,21	-14,89	-9,03
C.Oeste	1,64	1,29	1,45	-21,87	-11,87	1,82	1,6	1,61	-11,91	-11,3
**Brasil**	**1,76**	**1,32**	**1,33**	**-24,62**	**-24,27**	**1,64**	**1,44**	**1,48**	**-11,97**	**-9,55**
3° Quadrimestre										
Norte	0,49	0,44	-	-11,01	-	0,53	0,48	-	-8,26	-
Nordeste	1,24	1,15	-	-7,41	-	1,49	1,47	-	-1,19	-
Sudeste	2,18	1,71	-	-21,59	-	1,88	1,62	-	-13,69	-
Sul	3,61	2,3	-	-36,37	-	2,4	2,3	-	-4,2	-
C.Oeste	1,69	1,51	-	-10,57	-	1,9	1,35	-	-28,95	-
**Brasil**	**1,84**	**1,42**	-	**-22,89**	-	**1,64**	**1,45**	-	**-11,83**	-

A variação do gasto médio por hospitalização, considerando os períodos pré-pandêmico e pandêmico, é apresentada na Figura 3. No Brasil, o gasto médio por internação nos quadrimestres da pandemia foi maior do que nos quadrimestres análogos do ano de referência (2019). Esse padrão também foi identificado nas regiões Sul, Sudeste e Nordeste – a última teve como exceção apenas o terceiro quadrimestre de 2020, cujo gasto médio por internação foi menor do que no mesmo período em 2019. Norte e Centro-Oeste apresentaram comportamento destoante, com alguns quadrimestres do período de pandemia apresentando gasto médio por hospitalização maior do que no período pré-pandemia, mas outros com situação contrária (Figura 3).

**Figura 3:** Gasto médio por internação hospitalar por câncer bucal e de orofaringe (em reais), por quadrimestre, por macrorregião e para o Brasil, considerando período pré-pandemia (2019) e período pandêmico (2020 e 2021).

De modo geral, as análises que consideraram como referência o ano de 2019 apresentaram resultados semelhantes à análise de validação, que considerou como referência a média dos valores dos anos 2018 e 2019.

## DISCUSSÃO

Este estudo mostrou que as hospitalizações por CaB e CaOR no âmbito do SUS sofreram redução em todas as fases da pandemia no Brasil. A primeira e mais importante diminuição ocorreu no segundo quadrimestre de 2020, período no qual a pandemia começou a se acentuar no país, impelindo governos locais a instituírem medidas de afastamento social na tentativa de conter a chamada primeira onda. A partir de então – e até o final do período de estudo –, mesmo tendo havido períodos com redução menos expressiva do que a experimentada no segundo quadrimestre de 2020, as internações hospitalares por CaB e CaOR realizadas pelo SUS não mais retomaram o seu patamar pré-pandemia. A identificação de uma redução sustentada nessas taxas é um dos principais resultados desta análise. Ao investigar o impacto da pandemia sobre as internações hospitalares por CaB e CaOR no âmbito do sistema público de saúde do Brasil, esta investigação forneceu indícios da magnitude e da distribuição do problema e pode contribuir para o seu enfrentamento.

Por serem considerados de caráter essencial, os serviços hospitalares e de atenção ao câncer do SUS mantiveram-se em funcionamento durante a pandemia no Brasil. Contudo, a manutenção desses serviços não impediu que a crise sanitária desencadeada pela covid-19 afetasse a capacidade hospitalar referente a essa doença, conforme indicaram os resultados deste artigo e de pesquisas realizadas no período inicial da pandemia^11,17^. Possíveis razões para esse efeito são diversas e podem estar relacionadas à sobrecarga da estrutura hospitalar em decorrência do afluxo de casos de covid-19 que demandaram hospitalização, à preocupação em garantir leitos hospitalares para esses casos, à falta de profissionais, suprimentos e/ou equipamentos – esgotados ou realocados para a assistência à covid-19 –, aos reflexos sociais das medidas de distanciamento social e da crise econômica emergente da crise sanitária e ao receio dos próprios pacientes de se contaminarem pelo novo vírus, optando assim por não procurarem os serviços de saúde^18,19^, entre outras. Reduções na capacidade do sistema hospitalar para tratamento de casos de câncer de cabeça e pescoço em decorrência do cenário pandêmico foram relatadas também em outros países, como Índia, Espanha e Reino Unido^20^. Na Espanha, um estudo realizado em 44 serviços hospitalares indicou que 45,5% deles precisaram suspender cirurgias oncológicas consideradas de alta prioridade em pacientes com câncer de cabeça e pescoço em algum momento da pandemia^21^. Esses resultados, contudo, não são diretamente comparáveis aos deste estudo – apesar da semelhança do tema, os desfechos sob análise são distintos.

A redução das hospitalizações por câncer pode ainda estar refletindo, em parte, adiamentos de tratamento por decisões médicas, com o objetivo de equilibrar riscos, pois pacientes com câncer têm piores desfechos quando acometidos pela covid-19 do que a população em geral^23,24^. Contudo, especificamente para CaB e CaOR, essa não é a situação preconizada. Estudo de modelagem estatística estimou que, no contexto pandêmico, os casos de CaB e CaOR estariam entre os tipos mais favorecidos pelo tratamento imediato, em comparação com o adiamento^25^. Em recomendações atualizadas para o período da pandemia de covid-19, a European Society for Medical Oncology indica que tumores de boca e orofaringe em estadiamento T1 já devem ser considerados como alta/média prioridade para cirurgia primária e que todos os demais estadiamentos devem ser considerados como alta prioridade^26^. Entende-se, então, que a redução nas hospitalizações encontradas nesta análise não esteja refletindo essencialmente adiamentos programados de tratamento das neoplasias; os resultados podem estar retratando situações mais graves, como: (1) barreiras ao acesso a tratamento hospitalar durante a pandemia; e (2) impactos da pandemia no diagnóstico dessas doenças, com sub-identificação de casos que deveriam estar sendo admitidos na rede hospitalar para tratamento. Reduções na realização de procedimentos de diagnóstico de câncer bucal foram identificadas no Brasil^12,27,28^. A redução das hospitalizações em decorrência de subdiagnóstico é um cenário provável e inquietante, pois casos que deixaram de ser diagnosticados nesse período poderão chegar aos serviços hospitalares com lesões em estadiamento mais avançado, para as quais a chance de sobrevida é menor^5,13,14,29^.

Este estudo analisou as fases da pandemia por quadrimestres e, considerando a variação do número semanal de óbitos no país desde a notificação do primeiro caso (em fevereiro de 2020)^15^, assumiu que o primeiro quadrimestre de 2020 representou o período inicial da pandemia no Brasil; o segundo, representou a primeira onda; o terceiro, o primeiro período de atenuação e afrouxamento das medidas de distanciamento social; e, por fim, os dois quadrimestres de 2021 representaram a segunda onda da pandemia. A primeira e mais importante queda nas admissões hospitalares por CaB e CaOR no âmbito do SUS ocorreu na fase da primeira onda, período no qual o sistema de saúde enfrentou o primeiro colapso, com esgotamento de recursos materiais e humanos e sobrecarga estrutural em decorrência da intensa disseminação da covid-19 pelo país. Essa também foi a fase de implementação, pelos governos locais, das medidas mais restritivas de afastamento social^16^. As reduções identificadas na fase da primeira onda são, então, compatíveis com o caráter desordenado desse período. Contudo, apesar de haver discretas recuperações em relação à fase de primeira onda, nenhuma das fases seguintes exibiu retorno das taxas ao patamar pré-pandemia. A sustentação dessas reduções após mais de um ano do início da pandemia sugere que o impacto dessa crise sanitária na atenção hospitalar ao CaB e CaOR não foi uma situação momentânea, decorrente apenas de uma adaptação inicial da assistência a um contexto novo e atípico. O impacto dessa redução sustentada na mortalidade/sobrevida por essas neoplasias a curto e médio prazo ainda é incerto e precisa ser monitorado, em especial, porque se tratam de subtipos de câncer que, em tempos normais, já são demasiadamente letais – a sobrevida média em cinco anos é de 50%^5^.

Este estudo também identificou que a redução nas admissões hospitalares realizadas pelo SUS variou em magnitude nas diferentes macrorregiões do país. Norte e, sobretudo, Nordeste foram as regiões que apresentaram as menores reduções nas taxas de hospitalização. Já as regiões Sul e Sudeste apresentaram reduções maiores e constantes durante as diferentes fases da pandemia. Sul e Sudeste são as regiões do Brasil que apresentam melhores indicadores de acesso a serviços de saúde^30^, incluindo maiores taxas de realização de procedimentos para diagnóstico de câncer bucal^12^. Pela maior provisão de serviços de saúde, é provável que, nessas regiões, sejam diagnosticadas mais lesões malignas e, proporcionalmente, mais lesões em estadiamento inicial. Como o diagnóstico do câncer foi uma área bastante afetada pela pandemia no país, é possível que regiões que realizavam mais desses procedimentos manifestem maiores reduções nas hospitalizações. Ainda, entende-se que casos em estadiamento inicial têm menor chance de terem sido priorizados para tratamento pelo sistema de saúde em contexto de crise sanitária, visto que, diante de oferta limitada de estrutura hospitalar para tratamento, é esperado que casos mais avançados e urgentes sejam prioridade.

Sul e Sudeste exibiram um notável aumento do valor médio por internação por CaB e CaOR, a partir da fase da primeira onda – para as demais regiões, essa tendência não foi tão clara. Entende-se que Sul e Sudeste tenham mais possibilidade de organização do fluxo do cuidado em períodos de exiguidade de recursos, por meio de triagem seletiva, inclusive com uso de telemedicina, e de acompanhamento dos casos e de priorização de situações mais graves – e que necessitam de intervenções mais onerosas. Contudo, o padrão dos gastos nessas regiões também pode estar refletindo uma diminuição do diagnóstico de lesões em estadiamento mais inicial, que demandam tratamentos menos invasivos e custosos. Por fim, os procedimentos clínicos vinculados às internações diminuíram mais do que os procedimentos cirúrgicos em todas as fases da pandemia, a partir da fase de primeira onda. As orientações para manejo CaB e CaOR durante a pandemia não preconizaram substituições de cirurgia por outras modalidades de tratamento. Entende-se que os presentes resultados estejam refletindo o fato de que as demandas clínicas têm maior chance de corresponderem a uma necessidade eletiva e adiável ou manejável em ambiente ambulatorial do que as demandas cirúrgicas.

Este estudo apresenta uma série de limitações que devem ser consideradas na interpretação dos seus resultados. O SIH abrange as internações realizadas pelo SUS e não contabiliza as que ocorrem no âmbito da rede suplementar (hospitais privados e conveniados a seguros de saúde); com isso, este estudo não apresenta o panorama de todas as internações por CaB e CaOR ocorridas no país. Dados da Pesquisa Nacional de Saúde de 2019 indicam que cerca de 71,5% da população brasileira não tem posse de plano de saúde^31^, dependendo assim do SUS frente a necessidades de atendimento/hospitalização. Além disso, o câncer bucal é um desfecho associado a condições socioeconômicas: indivíduos em condições mais vulneráveis – e que, consequentemente, dependem mais dos serviços públicos de saúde – têm risco aumentado de desenvolver essa doença^32^. Assim, apesar de analisar apenas os dados vinculados ao SUS, entende-se que este artigo inclui parcela expressiva da população acometida por essa doença. Considerando o caráter universal do SUS e a impossibilidade de equacionar com precisão a proporção de pacientes com CaB e CaOR usuária de planos de saúde em âmbito hospitalar, destaca-se que as taxas apresentadas foram calculadas usando como denominador a população total.

As taxas de internação aqui apresentadas podem estar contabilizando mais de uma admissão hospitalar de um mesmo paciente pela mesma causa, pois o SIH disponibiliza os dados de modo consolidado e, sendo assim, essas duplicidades não podem ser identificadas e excluídas. Todavia, entende-se que essa não é uma limitação crítica para esta análise, que objetivou dimensionar reduções na prestação de serviços hospitalares possivelmente associadas à pandemia, sem considerar aspectos relativos a riscos individuais. Ainda, pontua-se que a opção de análise dos dados por macrorregião e usando os quadrimestres para representar as fases da pandemia – com padronização dessa divisão para todas as regiões – desconsidera a heterogeneidade nos padrões espaço-temporais de disseminação que esteve presente no transcorrer da pandemia no Brasil. Futuros estudos que considerem características de ordem local da dinâmica da pandemia no país podem ser oportunos.

Este artigo revelou que, após mais de um ano do início da pandemia no Brasil, a rede hospitalar de cuidado ao CaB e CaOR do SUS ainda não tinha se restabelecido. Também apresentou indícios de que, durante a pandemia, a rede hospitalar priorizou casos mais graves de CaB e CaOR. As internações que deixaram de ser realizadas podem estar refletindo barreiras ao acesso a tratamento hospitalar, pela sobrecarga e desestruturação desse setor em decorrência da pandemia. A demanda reprimida por hospitalizações para casos de CaB e CaOR, que são doenças de rápida evolução, estará (e já está) relacionada aos atrasos para início do tratamento, com impacto negativo para a sobrevida desses pacientes – situação que se agravará proporcionalmente ao tempo que durar essa desestruturação. Este estudo destaca a necessidade de atenção da gestão em saúde para a rede de cuidado ao CaB e CaOR, pois esta, além da necessidade imediata de retomada da sua capacidade de atenção, provavelmente precisará ampliá-la, já em curto prazo, para mitigar os prováveis danos da pandemia no cuidado a essas doenças e evitar um reflexo dessa disrupção no aumento da mortalidade.
